# Development of an *in vitro PIG-A* gene mutation assay in human cells

**DOI:** 10.1093/mutage/gew059

**Published:** 2017-01-05

**Authors:** Benjamin J Rees, Matthew Tate, Anthony M Lynch, Catherine A Thornton, Gareth J Jenkins, Richard M Walmsley, George E Johnson

**Affiliations:** 1 Institute of Life Science, Swansea University Medical School, Singleton Park, Swansea, UK; 2 Gentronix Ltd BioHub at Alderley Park, Alderley Edge, Cheshire, UK; 3 GlaxoSmithKline R&D, Ware, Hertfordshire, UK; 4 Faculty of Life Sciences, University of Manchester, Manchester, UK

## Abstract

Mutagens can be carcinogens, and traditionally, they have been identified *in vitro* using the *Salmonella* ‘Ames’ reverse mutation assay. However, prokaryotic DNA packaging, replication and repair systems are mechanistically very different to those in the humans we inevitably seek to protect. Therefore, for many years, mammalian cell line genotoxicity assays that can detect eukaryotic mutagens as well as clastogens and aneugens have been used. The apparent lack of specificity in these largely rodent systems, due partly to their mutant p53 status, has contributed to the use of animal studies to resolve data conflicts. Recently, silencing mutations at the *PIG-A* locus have been demonstrated to prevent glycophosphatidylinositol (GPI) anchor synthesis and consequentially result in loss of GPI-anchored proteins from the cell’s extracellular surface. The successful exploitation of this mutant phenotype in animal studies has triggered interest in the development of an analogous *in vitro PIG-A* mutation screening assay. This article describes the development of a robust assay design using metabolically active human cells. The assay includes viability and cell membrane integrity assessment and conforms to the future ideas of the 21st-century toxicology testing.

## Introduction

Genetic toxicology plays an essential role within hazard identification and risk assessment during the development of novel drugs as well as pesticides, herbicides, flavours, and fragrances. Throughout the early stage drug development, a substance’s ability to damage DNA through genotoxic mechanisms must be fully investigated to enable accurate and cost-effective hazard and risk assessment (1). If possible, this would be completed with more emphasis on high content, high throughput *in vitro* genotoxicity assessment, reducing animal usage. Short falls in *in silico* pharmacokinetic and dynamic modelling (2)] as well as reportedly poor *in vitro* specificity in carcinogenicity prediction (3) have produced a battery of *in vitro* and *in vivo* genotoxicity assays developed to identify potential mutagens, aneugens and clastogens (4,5), which could benefit from a broader revision to include 21st-century approaches.

Several regulatory-accepted *in vitro* mammalian cell mutation assays are available to assess chemically induced gene mutation. These use cell lines derived from mice (L5178Y) and hamster (CHO, AS52 and V79), which are often p53 mutant, and humans (TK6) (6). The most commonly used genetic endpoints are mutation at the thymidine kinase (*TK*) and hypoxanthine–guanine phosphoribosyl transferase (*HPRT*) loci. Whilst data from the *HPRT* and *TK* mutation tests are widely accepted in hazard and risk assessment (6), they are relatively time-consuming (3–6 weeks) and highly labour-intensive, particularly when characterising dose–response relationships, and reportedly have poor specificity (3) that can limit their utility in a screening context. However, specificity issues are being addressed by a more recent focus on p53 competent human cell lines within *in vitro* Organisation for Economic Cooperation and Development (OECD) guidance documents (7). To date, *in vivo* gene mutation experiments have been restricted largely to transgenic models (MutaMouse™ and BigBlue). As these are more expensive than inbred animals, they are only used in a regulatory setting as a study of ‘last resort’, addressing specific concerns about a potential mutagenic signal (identified *in vitro*) and/or mechanism.

Recently, a novel gene mutation assay based on the endogenous phosphatidylinositol–glycan biosynthesis class-A (*PIG-A*) gene (8), located on the *p* arm of the X-chromosome (9) was developed in rodents (10). *PIG-A* encodes an enzyme critical to the synthesis of glycophosphatidylinositol (GPI) anchor molecules (11,12). Specifically, *PIG-A* is essential in the production of a catalytic subunit of the *N*-acetylglucosamine transferase complex (13), and combined with numerous other *PIG*-gene products, *pig-b*, *pig-c* etc., it contributes to the synthesis of the final branched glycan structure of the anchor. This eventually resides on the external surface of the cellular membrane, extending into the extracellular space, tethering cell-specific and conserved surface antigens (14). Whilst silencing mutations in any of these genes may prevent GPI anchor synthesis (15,16), a mutational silencing event within *PIG-A* is believed to be the most common cause of GPI anchor synthesis disruption, because it is X-linked (17), and a single mutation can result in a deficiency of GPI-anchored cell surface antigens. Hence, the GPI anchor-deficient phenotype is generally attributed to *PIG-A* mutation (18). The mutant genotype (*PIG-A*^−^) is also a characteristic of patients with paroxysmal–nocturnal–haemoglobinuria (PNH) (19), and diagnostic methods have been developed to detect and quantify cells carrying presumptive mutations at the *PIG-A* locus using flow cytometry (FCM) (20). The phenotype is reported to be growth neutral *in vivo* (21), an important factor in mutagenesis studies as it avoids mutational bias. Mutant frequency (*f*) and mutation rate (*µ*) at the *PIG-A* locus can be measured indirectly, using FCM, recording the loss of expression of specific GPI-anchored cellular antigens following mutagen exposure (20,22). The assay potentially has great transferability between mammalian species, due to the highly conserved nature of GPI-anchor synthesis (23).

The development of the rodent erythrocytic *in vivo Pig-a* gene mutation assay has gathered significant momentum, benefitting from extensive coordinated ring trials (24–27), methods to support assay transfer across mammalian species (21,23,28–34) and high throughput optimisation (29). In addition, there has been some progress in demonstrating the mechanistic basis of the assay (32,35,36), and efforts are going on to further characterise the assay in terms of genotoxic mechanisms (37,38) and chemical space. It is hoped that these activities will support the development of an OECD guideline in due course. Following recent European Union reforms to limit and/or ban animal testing (39), especially relevant to the cosmetics and consumer industries (40,41), there has been increased focus on replacing animal testing with novel approaches to quantify genotoxic hazard for human risk assessment purposes. Innovative technologies are being developed to enable high throughout, high content screening whilst retaining a high level of sensitivity (42–45).

As part of these efforts, our laboratories have focused efforts on the development of an *in vitro PIG-A* assay design that could be used within a screening or a regulatory setting and serve as a predictive counterpart to the *in vivo* assay described above. TK6 was initially proposed as the cell line of choice based on the following criteria: (i) a non-adherent cell line of haemopoetic origin and short cell cycle time to allow short experimental duration; (ii) a cell line with a proven track record of high sensitivity and specificity in genotoxicity assays; and (iii) a cell line of human origin (with functional p53) to improve risk assessment, species specificity, and with the potential for future *in vitro* to human extrapolation. This decision was further supported by a recent review of cell lines used in genotoxicity testing, which had preferentially highlighted the benefits of TK6 cells (46–48). Other laboratories have described the development of an analogous *in vitro PIG-O* assay in DT40 chicken cells (49) and an analogous *in vitro PIG-A* assay in TK6 cells (42). These assays theoretically provide the means for more extensive mechanistic validation (38), though for wide acceptance, the phenotypic data will need DNA sequence data to unambiguously link phenotype to genotype.

This study reports development of a *PIG-A* mutation assay in human B-lymphoblastoid cell lines. The aim was to establish a robust assay platform that takes into account the potential limitations of the system. Initially, enrichment techniques were evaluated to enable the assessment of GPI anchor surface proteins CD59 and CD55 (see supplementary data, available at Mutagenesis Online) as potential reporters of phenotypic *PIG-A* mutation. The need for this reflects early findings that the prevalence of the loss of GPI anchor phenotype in TK6 cells was very much higher than the expected mutation frequency. In addition, aerolysin sensitivity was used to validate *PIG-A* phenotype and was also explored as a potential basis for mutant selection, useful in tying phenotype to genotype. Dose–response data were generated throughout and were used to investigate the sensitivity of the current *PIG-A* assay design to both genotoxic and non-genotoxic compounds, with confocal microscopy utilised to provide additional molecular confidence in the phenotype. Throughout development, the assay design incorporated viability assessments, comprehensive gating strategies as well as a marker for non-GPI–associated cell surface protein markers, a potential source of misleading results. During the course of the study, the alternative, metabolically active human MCL-5 cells were found to provide a lower background spontaneous mutant prevalence. These assay design advancements with the associated flow cytometric gating strategies suggest development of an improved assay platform and the basis for further optimisation and validation.

## Materials and methods

### Cell culture

The TK6 cell line was obtained from the American Type Culture Collection (ATCC; http://www.atcc.org/). Low passage TK6 cells were obtained from Masamitsu Homna (NIH, Japan). AHH-1 and MCL-5 cell lines were purchased from Gentest Corporation (now BD Biosciences, Oxford, UK). TK6, AHH-1 and MCL-5 cells were cultured in 89% RPMI 1640, 1% l-glutamine (Gibco®, Paisley, UK) and 10% horse serum (BioSer, Sussex, UK): MCL-5 culture media were supplemented with 50 mg/mL Hygromycin B (Merck, Hoddesdon, UK). Low passage TK6 growth medium consisted of 89% RPMI 1640, 1% penicillin–streptomycin (Gibco®, Paisley, UK) and 10% heat-inactivated horse serum (cat no. 26050-088, Gibco). All cultures were kept within a controlled carbon dioxide (CO_2_; 5 ± 1%) incubator maintained at 37.5°C. Prior to use, cryovials of human lymphoblastoid TK6, low passage TK6, MCL-5 and AHH-1 cells were removed from cryopreservation, thawed quickly and cultured under standard conditions. All cell lines were monitored regularly to detect signs of contamination (microscopy) or morphological abnormalities and subcultured once prior to the experimental start date to ensure reproducible growth dynamics. Stock cell cultures were vitally maintained between 10^5^ and 10^6^ cells/mL in 75 cm^2^ culture flasks by routine subculturing, with an exception of the TK6 cell line, which was able to grow consistently from a 4 × 10^5^ cells/mL seeding concentration. Populations depleted of *PIG-A* mutant cells were cryopreserved and stored in liquid nitrogen for a period of up to 6 months. To revive cells from cryogenic storage, they were thawed and cultured for a period of at least 72 h prior to use. Where appropriate, the use of fluorescently labelled isotype control antibody samples were utilised in order to determine the nature of cell line-specific antibody binding, thereby reporting accuracy.

### Enrichment strategies to optimise CD59 expression within TK6 cells

#### Dynabead® magnetic bead retrieval

A mouse anti-human CD59 antibody (cat no. 555761, BD Pharmingen™, San Jose, CA, USA) was biotinylated (DSB-X™ Biotin Protein Labelling Kit, cat no. D-20655, Molecular Probes, Life™ Technologies, Paisley, UK) and used with the Dynabeads® Flow Comp™ Flexi Kit (cat no. 11061D, Molecular Probes, Life™ Technologies, Paisley, UK) to select cells expressing CD59, i.e. the cells with a *PIG-A* wild-type phenotype. Briefly, 5 × 10^7^ TK6 cells were resuspended in 500 µL of 2–8°C isolation buffer and 25 µL of DSB-X™ biotin-labelled antibody, mixed well and incubated at 2–8°C (protected from light) for 10 min. Cells were then washed (2 mL cold isolation buffer), collected by centrifugation (350 × *g* for 8 min) and the supernatants aspirated prior to resuspension in 1 mL cold isolation buffer and the addition of 75 µL Flow Comp™ Dynabeads®. Following mixing, the suspension was incubated for 15 min (2–8°C, rolling and tilting), then transferred into a 2-mL microcentrifuge tube and placed on a magnetic rack (Qiagen) for 2 min. Following removal of the supernatant, the tube was then detached from the magnet and the pellet of bead-bound cells was washed, followed by resuspension in 1 mL of cold isolation buffer and then re-pelleted (twice). The bead-bound cells were then resuspended in 1 mL of Flow Comp™ release buffer for 10 min (25°C under rolling and tilting) and then placed on the magnetic rack for 2 min. The supernatant containing the bead-free cells was transferred into a new tube and placed on the magnetic rack for an additional 2 min to remove any residual Dynabeads®. Finally, the supernatant was centrifuged (350 × *g* for 8 min), and the resulting enriched population of cells expressing CD59 was transferred to a 25 cm^2^ cell flask for culture. Quality assurance (QA) FCM based on the *PIG-A* assay was used to determine the level of CD59 enrichment in the cell culture (10^6^ cells were removed and stained, see Determination of *PIG-A* mutant frequency using the CD59 phenotypic marker following exposure to the potent mutagen EMS, to determine the frequency of CD59-positive cells, i.e. a *PIG-A* wild-type phenotype).

#### Clonal expansion

Cultures were established from cryopreserved TK6 cell stocks and analysed using FCM to determine the proportion of cells expressing the CD59 antigen, by means of the anti-human CD59-R-PE–labelled antibody. >10 × 10^6^ cells were then harvested by centrifugation (250 × *g* for 7 min) and resuspended to a final concentration of 200 cells/mL prior to clonal expansion as follows: 100 μL cellular samples were transferred into each well of six identical 96 well plates (Greiner–Bio-One, Gloucestershire, UK), and following 72 h incubation, each well was inverted microscopically assessed for colony formation. From these plates, a total of 144 deemed well-established colonies were transferred into six 24-well plates, with the addition of fresh growth medium (~300 µL), and the cells were cultured for a further 72 h. The 10 largest colonies (excess of ~1000 cells) were transferred into separate sterile 25 cm^2^ flasks and cultured to confluency (~1–7 × 10^5^ cells/mL) within pre-warmed culture media. Samples were subsequently analysed for CD59 expression by FCM, and the cultures with the resulting highest frequency of cells expressing CD59 were prepared for long-term cryopreservation.

#### Fluorescence-activated cell sorting

A fresh vial of TK6 cells homologous to the cultures used for the clonal expansion experimentation, subcultured from the original TK6 cell stock, were analysed for CD59 surface antigen expression using anti-human CD59-R-PE labelling as well as human leucocyte antigen–antigen DR (HLA-DR) presence utilising an anti HLA-DR fluorescein isothiocyanate (FITC) antibody and subsequent FCM. Approximately 20 000 cells that were CD59-positive and displayed uniform HLA-DR fluorescence were isolated using fluorescence-activated cell sorting (FACS; sorting on a FACSAria 1, BD Biosciences, UK). The recovered cells were pooled into 5 mL sorting tubes (BD Biosciences, Hertfordshire, UK) within culture medium, pelleted and cultured in fresh culture media within 25 cm^2^ culture flasks prior to long-term cryopreservation. Once more QA FCM analysis was performed to confirm the frequency of CD59-positive cells, i.e. a *PIG-A* wild-type phenotype following enrichment and prior to preservation.

### Determination of *PIG-A* mutant frequency using the CD59 phenotypic marker following exposure to the potent mutagen EMS

For this purpose, 2 × 10^6^ clonally enriched TK6 cells for CD59-positive expression (see Enrichment strategies to optimise CD59 expression within TK6 cells–Clonal expansion) were thawed and cultured under standard conditions. Following sufficient growth, 10^5^ cells/mL were seeded into 12.5 cm^2^ culture flasks and treated with either vehicle [dimethyl sulphoxide (DMSO) 1% v/v] or ethylmethane sulfonate (EMS; 0–10 µg/mL) for 24 h at 37°C, 5% CO_2_. Following treatment, the cells were washed [10 mL phosphate-buffered saline (PBS)] and cell density determined (Beckman Coulter Z1 Particle Counter). Then, 10^6^ cells were removed, washed (10 mL PBS) and centrifuged (250 × *g* for 7 min) prior to resuspension in 50 µL of fresh RPMI-based culture medium (see Cell culture). Simultaneously, parental-treated cell cultures were subcultured to 10^5^ cells/mL and incubated for a further 24 h to enable subsequent daily *PIG-A* mutation assessments, only if suitable population growth has occurred to enable this (>2 × 10^5^ cells/mL). The resuspended cellular samples were then incubated with 20 µL (0.1 mg/mL) of anti-human CD59 PE antibody for 20–30 min at 2–8°C (protected from light). Immediately following this, the cells were harvested by centrifugation (250 × g for 7 min), the supernatant was carefully removed and the pellet washed in 10 mL PBS. This was followed by re-pelleting and further resuspension in 1 mL PBS prior to being directly transferred into 5 mL BD Flow Tubes (protected from light). A portion of the cells was analysed by FCM (BD FACS Aria I) daily for four additional experimental days following the determination of spontaneous background mutant frequencies on Day 0. Mutant phenotype gating was subsequently undertaken following the use of an unstained instrument calibration standard in order to establish an autofluorescence level within the channel and hence set the detector voltages correctly to enable a fluorescence threshold value of negativity. Once established, subsequent stained samples were able to be accurately assessed for the reporting fluorophore of GPI-anchored protein (AP) status.

### Investigations of FCM defined *PIG-A* phenotypes within TK6 cell populations

#### Aerolysin permeabilisation

First, 2 µM pro-aerolysin (AeroHead Scientific, Saskatoon, Canada) was activated with 2 µg/mL trypsin (diluted in 25 µg/mL Tris pH 8 buffer) in a 1:1 solution to produce a 250 nM aerolysin solution. Then, parental TK6 *PIG-A* wild type and *PIG-A* mutant populations (isolated by FACS) were adjusted to 2 × 10^5^ cells/mL in RPMI 1640 and treated with a range of aerolysin concentrations (15.625–250 nM) in RPMI 1640. After 48 h, 150 µL cell cultures were removed from each well and viability assessed using FCM and propidium iodide (PI) DNA staining: viable cells are PI-negative, and permeabilised non-viable cells are PI-positive; PI was detected by increased fluorescence in the FL3-H detector on the FACSCalibur (Cell Quest Pro version 5.2.1).

#### Cell viability assessment

First, 10^6^ untreated TK6 cells, clonally enriched for the CD59 surface marker, were resuspended in 50 µL of fresh culture medium and then incubated with 20 µL (0.1 mg/mL) of anti-human CD59 R-PE antibody for 20–30 min at 2–8°C (protected from light). Immediately following this, cells were harvested by centrifugation (250 × g for 7 min), the supernatant was removed and the pellet washed in 2 mL PBS to remove residual unbound antibody. Simultaneous to the washing steps, 1X Annexin V-binding buffer was prepared in deionised water (dH_2_0) and placed on ice to cool. Following a second 2mL PBS wash, cells were resuspended in 2 mL 1X Annexin V-binding buffer, centrifuged and resuspended in an additional 100 µL of the 1X Annexin V-binding buffer. Then, 5 μL of Alexa Fluor® 488 Annexin V conjugate was added to the 100 µL cell suspension and incubated at room temperature for 15 min, protected from light. Post incubation, 2 mL 1X Annexin-binding buffer was added to each sample, centrifuged (250 × g for 7 min) and resuspended in a further 200 µL of 1X Annexin-binding buffer. Finally, 5 µL (0.25 μg) of 7-aminoactinomycin D (7-AAD) solution was added to each sample, incubated for a minimum 15 min on ice and subsequently diluted via the addition of 1–2 mL of PBS and transferred directly into 5 mL BD Flow Tubes, protected from light, and analysed within 4 h of initial antibody treatment.

### GPI-AP candidate trials in different human cell lines

#### Fluorescent aerolysin

Prior to use, 1 mL of PBS was added to 25 μg of powdered (lyophilised) fluorescent aerolysin (FLAER); this stock solution was further diluted 1 in 10 (dH_2_0) and 50 μL volumes aliquotted into 0.5 mL microcentrifuge tubes for long-term storage (wrapped aluminium foil, −20°C). Samples for each cell line, containing 10^6^ cells in suspension, were transferred into sterile 15 mL plastic disposable tubes, centrifuged (250 × g for 7 min) and resuspended in 100 μL of fresh pre-warmed culture medium (see Cell culture). Cell pellets were carefully redispersed and treated with 5 μL of FLAER (in triplicate) at 3 concentrations; 1×, 0.5× and 0.1× in 2% bovine serum albumin (BSA)/PBS. Each tube was incubated for 15 min at room temperature and protected from direct light at all times. Post incubation, samples were centrifuged, washed with 10 mL PBS and resuspended within a further 1 mL of PBS. All samples were placed on ice and protected from direct light to maintain the stability of the suspensions and the fluorophores used, prior to flow cytometer acquisition.

#### CD48–B-Lymphocyte Activation Marker 1 and CD55/59 staining protocol

Samples for each cell line (10^6^ cells) were pelleted and resuspended in fresh medium (100 µL) and fluorescently labelled with either 20 μL of mouse anti-human anti-CD48 R-PE antibody (cat no. 552855, BD Biosciences, Hertfordshire, UK) or with 20 μL of mouse anti-human anti-CD55 R-PE antibody and 20 μL of mouse anti-human anti-CD59 R-PE antibody (room temperature, protected from direct light, 30 min). The cells were pelleted (250 × g for 7 min), washed in 2 mL PBS and resuspended in a further 1 mL PBS and placed on ice, protected from light, prior to flow cytometer analysis.

### Viability assessment of MCL-5 Cells to avoid potential Artefacts

First, 100 μL of 1 μM Staurosporine solution was added to six separate MCL-5 cellular suspensions (4 × 10^6^ cells in 10 mL) and incubated for either 30 min, 1 h, 2 h, 3 h, 4 h or 8 h at 37°C. Cells were pelleted (250 × g for 7 min), washed twice in 10 mL PBS and resuspended in a further 10 mL of fresh medium. Following each treatment, cellular densities were determined, and three further cellular samples were constructed each containing 10^6^ cells. The samples were then stained with anti-human anti-CD55 R-PE, CD59 R-PE, 7-AAD and Annexin V in an analogous protocol to that as described above.

### Determination of *PIG-A* mutant frequency using the CD59 phenotypic marker, viability assessment and HLA-DR staining within MCL-5 cells

MCL-5 cellular cultures were established from previously cryopreserved parental stocks. Cultures were exposed to either EMS (0–161.1 µM), *N*-methyl-*N*-nitrosourea (MNU; 0–80.5 µM), *N*-ethyl-*N*-nitrosourea (ENU; 0–322.2 µM) or 2,4 dinitrophenol (2,4 DNP; 0–325.9 µM) with DMSO being utilised and as a vehicle control for 24 h. Following treatment, cultures were washed (10 mL PBS), counted, seeded (for subsequently daily analysis) and 10^6^ cellular samples stained ready for initial flow cytometric analysis; 20 µL anti-human anti-CD59 R-PE, 40 μL HLA-DR FITC (cat no. 555871, BD Biosciences, Hertfordshire, UK) and 10 μL 7-AAD were used in an analogous fashion to previous protocols. Flow cytometric acquisition (FACSCalibur (Cell Quest Pro version 5.2.1)) was performed within an hour of sample preparation, and samples were stored on ice protected from direct light beforehand. The experiment was carried out daily for four additional days following the determination of the spontaneous background mutant frequencies on Day 0.

### Tandem GPI-AP confocal expression analysis in MCL-5 cells

Untreated MCL-5 cells (10^6^) were stained with 20 µL anti-human anti-CD55 and/or 20 µL CD59 R-PE fluorescent antibodies for 30 min, washed twice in 3 mL pre-warmed (37°C) PBS and resuspended and fixed in 1 mL of 4% PBS-buffered paraformaldehyde for 15 min at 37°C, before being washed twice more with 3 mL PBS. Coverslips, precision 0.17 mm thickness, (cat no. 474030–9000, Carl-Zeiss, Hertfordshire, UK) were placed in a 0.1-mL aliquot of 1mg/mL aqueous poly-l-lysine (Sigma-Aldrich, Gillingham, UK) on parafilm, incubated for 10 min at room temperature, washed thoroughly using dH_2_0 and left to air-dry. Coverslips were then incubated in excess 1% BSA (Sigma-Aldrich, Gillingham, UK) PBS for at least 1 h, washed and placed into 6-well plates (Greiner Bio-One, Hertfordshire, UK). Three millilitre of stained cell suspension, at a suitable concentration to provide sufficient coverage ~10^5^–10^6^ cells/mL, was added to each well to enable cellular adhesion and incubated for 10 min at room temperature, protected from light. Following incubation, each coverslip was microscopically inspected to ensure that attachment was successful before being further washed in 3 mL PBS and carefully touch dried. Coverslips were then mounted within 1,4-diazabicyclo[2,2,2]octane (DABCO) (cat no. D27802, Sigma Aldrich, Gillingham, UK); 45 mL glycerol (G55-16 100 mL, Sigma Aldrich, Gillingham, UK), 2.5 mL Tris-hydrochloric acid buffer (pH 8) and 2.5 mL dH_2_0. Polished glass slides (cat no. 11562203, Fisher Scientific, Loughborough, UK) with autoclave tape were used to construct posts, which loaded coverslips were laid down onto 50 μL DABCO, centrally between the two posts. Confocal microscopy was carried out using an LSM-710 laser scanning confocal microscope and supplied ZEN software version 2009 in conjunction with either a 40× or 63× oil immersion DIC objective (Carl Zeiss, Cambridge, UK).

### Gating strategy investigations

First,10^6^ untreated MCL-5 cells were stained with 20 µL anti-CD55/CD59 R-PE antibodies and washed (3 mL PBS) prior to flow cytometric analysis, as described previously. Samples were run/tested in triplicate utilising the different proposed gating strategies; typically FSC-A and SSC-A scatter plots were utilised to define a typically ‘single-cell’ population, from these outputs, i.e. 2-D histograms and 2-D density scatter plots were constructed utilising the FL-W measure of the fluorophore channel (PE) as well as the FSC-W, as proposed measures of particle size. Gates were drawn, which encompassed at least 90% of the parental population, to further isolate particles defined ‘single cell’ in nature and finalised outputs were commented in terms of numbers/percentage of single cell CD55/59-positive phenotypic *PIG-A* mutant events, as described above. All these parameters were managed using the FACS Diva™ 6 software, and doublet discrimination plots were removed to enable easier visualisation of results.

In addition, MCL-5 cellular cultures were established from previously cryopreserved parental stocks. Cultures were exposed to MNU (0–1 µg/mL) with DMSO being utilised and as a vehicle control for 24 h. Following treatment, cultures were washed (10 mL PBS), densities defined, seeded (for subsequently daily analysis) and 10^6^ cellular samples stained ready for initial flow cytometric analysis; anti-human anti-CD59 and anti CD55 R-PE, Annexin V and 7-AAD were used in an analogous fashion to previous protocols. Flow cytometric acquisition was undertaken on a BD FACSAria 1 (FACSDiva™ 6) and performed within an hour of sample preparation, and samples were stored on ice protected from direct light beforehand.

## Results and Discussion

### Enrichment strategies to optimise CD59 expression within TK6 cells

Immunofluorescence antibodies to a number of GPI-anchored surface antigens (CD48 and CD59, see Supplementary figure 1, available at Mutagenesis Online) revealed that the parental TK6 cell line comprises a heterogeneous population, with a significant minority of cells GPI-anchored protein deficient. Mutagenicity is measured as an increase over the spontaneous (pre-existing) background of mutant cells, so a high background poses a significant barrier to the development of an assay to detect rare mutation events. Consequently, strategies to deplete parental TK6 cultures of the presumptive, pre-existing mutant populations are required (50). The regulatory accepted MLA and HPRT *in vitro* mammalian gene mutation assays have analogous requirement to purify the pretest cell cultures of mutants and subsequently enrich for wild-type cells. However, a different strategy was required for analysis of *PIG-A*; three approaches were assessed, immunomagnetic bead separation (Dynabead®), which is already used to increase statistical power in the *in vivo Pig-a* assay (29), clonal expansion and FACS. Each method was compared for its ability to produce near-homogenous cell populations with low phenotypic *PIG-A* mutant prevalence.

All three enrichment methods reproducibly demonstrated >90% homogeneous GPI-AP (CD59)-positive populations (Table 1). FACS produced the optimum results in terms of average CD59 wild-type enrichment, 95.3% ± 3.0, with Dynabead® and Clonal expansion yielding 89.1% ± 6.5 and 70.8% ± 25.3 CD59-positive cell cultures respectively. In terms of reproducibility, FACS and Dynabead® were shown to be the most robust methodologies, with clonal expansion resulting in a much greater range of purities post enrichment as emphasised by the elevated spread of data around the mean. Clonal expansion did however result in the optimum individual enrichment purity, with 99.8% of the subsequent population positive for CD59 expression.

**Table 1.** Phenotypic *PIG-A* assessment of TK6 populations following enrichment

	Sample ID	Number of total events	CD59-positive (%)	CD59-negative (%)	SD
Dynabead	1a	8216	84.1	15.9	
	1b	8461	84.3	15.7	
	1c	8379	84.5	15.5	
	2	11388	96.5	3.5	
	3	10000	95.9	4.1	
	Average	9288.8	89.1	10.9	6.5
Clonal Expansion	1	9427	99.8	0.2	
	2	10170	84.5	15.5	
	3	9767	54.7	45.3	
	4	9836	99.7	0.3	
	5	10080	28.0	72.0	
	6	9679	61.8	38.2	
	7	9708	54.4	45.6	
	8	10443	83.8	16.2	
	Average	9888.8	70.8	29.2	25.3
FACS	1a	9480	95.5	4.5	
	1b	9467	96.2	3.8	
	1c	9337	95.6	4.4	
	2a	9867	97.7	2.3	
	2b	10179	95.9	4.1	
	2c	10194	87.6	12.4	
	3a	10900	96.1	3.9	
	3b	9919	96.5	3.5	
	3c	9787	96.3	3.7	
	Average	9903.3	95.3	4.7	3.0

Anti-CD59 R-PE antibody utilised in conjunction with flow cytometry to assess GPI-AP percentage expression within Dynabead®, Clonal Expansion and FACS-enriched populations of cells (~10 000 single cellular events captured).

Within the three trialled methodologies, Dynabead® and FACS were demonstrated to be highly robust, reproducible methods; however, they lacked the refinement to achieve the prerequisite sensitivity. In the case of Dynabeads®, the technology favours the use of antigens with larger molecular weights and FACS, especially prevalent within older cell sorters, is highly dependent upon operator ability, machine usage and cellular viability. Even though clonal expansion was demonstrated the most inconsistent in terms of enrichment efficiency, the method generated the optimum enrichment yield (99.8%)—the lowest background phenotypic *PIG-A* mutant frequency (~2000 × 10^6^) and was therefore used for preliminary proof of principle experiments.

### Determination of *PIG-A* mutant frequency using the CD59 phenotypic marker following exposure to the potent mutagen EMS

**Figure 1.**
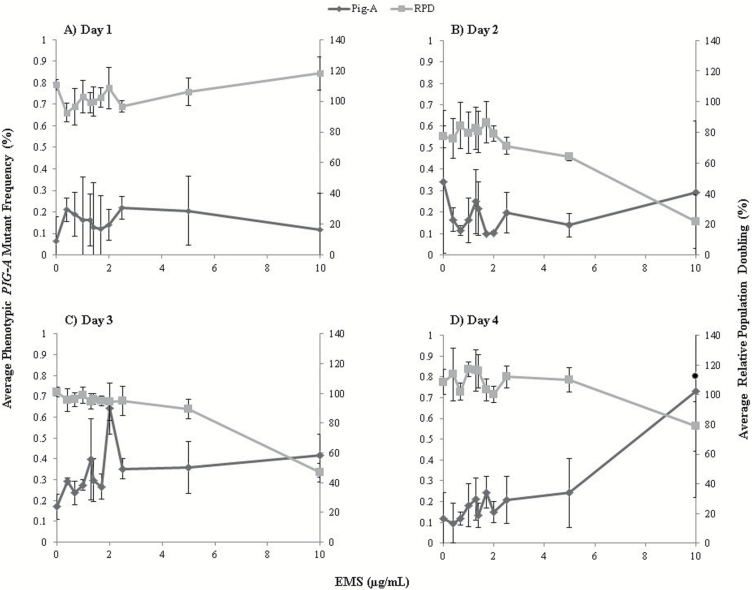
Human lymphoblastoid cells (TK6) following 24 h EMS exposure, CD59 antigen utilised as phenotypic *PIG-A* marker. Day 1–4 frequencies of *PIG-A* mutant TK6 cells (clonally selected) following 24 h low-dose EMS exposure—CD59 antigen utilised as reporter of mutation (Dunnett’s test; *P < 0.05; *n* = 3, error bars ±1 SD; 0.1% *PIG-A* mutants = 1 mutant × 10^–3^ cells).

In order to assess the sensitivity of the proposed assay design, the performance of CD59 as a phenotypic reporter for *PIG-A* mutation was evaluated in clonally enriched TK6 cell cultures (TK6^CD59+^) exposed to a low-dose range of EMS (Figure 1).

A statistically significant increase in the frequency of cells negative for CD59 staining at Day 4 of analysis was observed with a 6-fold increase compared with concurrent controls (*P* < 0.01, Dunnett’s test). Prior to Day 4 analysis, there were no statistically significant increases in CD59-negative cells at any dose, providing evidence for a time-dependent induction of the mutant phenotype, apparently consistent with mutation induction. The lowest observed genotoxic effect level was defined as 10 µg/mL, with minimal cytotoxicity observed [~80% relative population doubling (RPD)] at the top dose.

Mathematical modelling (PROAST38.9 software) was used to further characterise the phenotypic *PIG-A* mutant dose response with EMS. Benchmark dose BMD_10_ and BMDL_10_ values (51) of 0.673 and 0.503 µg/mL, respectively, were observed with comparable BMD values for EMS to those reported by Doak *et al*. (52) in the *HPRT* cell mutation assay (53). However, because the spontaneous phenotypic mutant frequency (~0.2%) in controls was high, additional studies were conducted to further investigate the relationship between the absence of extracellular GPI-AP staining and *PIG-A* mutation (Supplementary Figures 2 and 3, available at Mutagenesis Online).

### Investigations of FCM defined *PIG-A* phenotypes within TK6 cell populations

Aerolysin permeabilisation has previously been reported as a means to confirm a *Pig-a* mutant phenotype (15,17,54). Aerolysin is a bacterial toxin secreted by *Aeromonas hydrophila,* which causes GPI-anchor–dependent cell lysis (54–56). Therefore, aerolysin is a GPI-anchor–specific toxin that can be used to demonstrate *PIG-A* mutant cell resistance.

**Figure 2.**
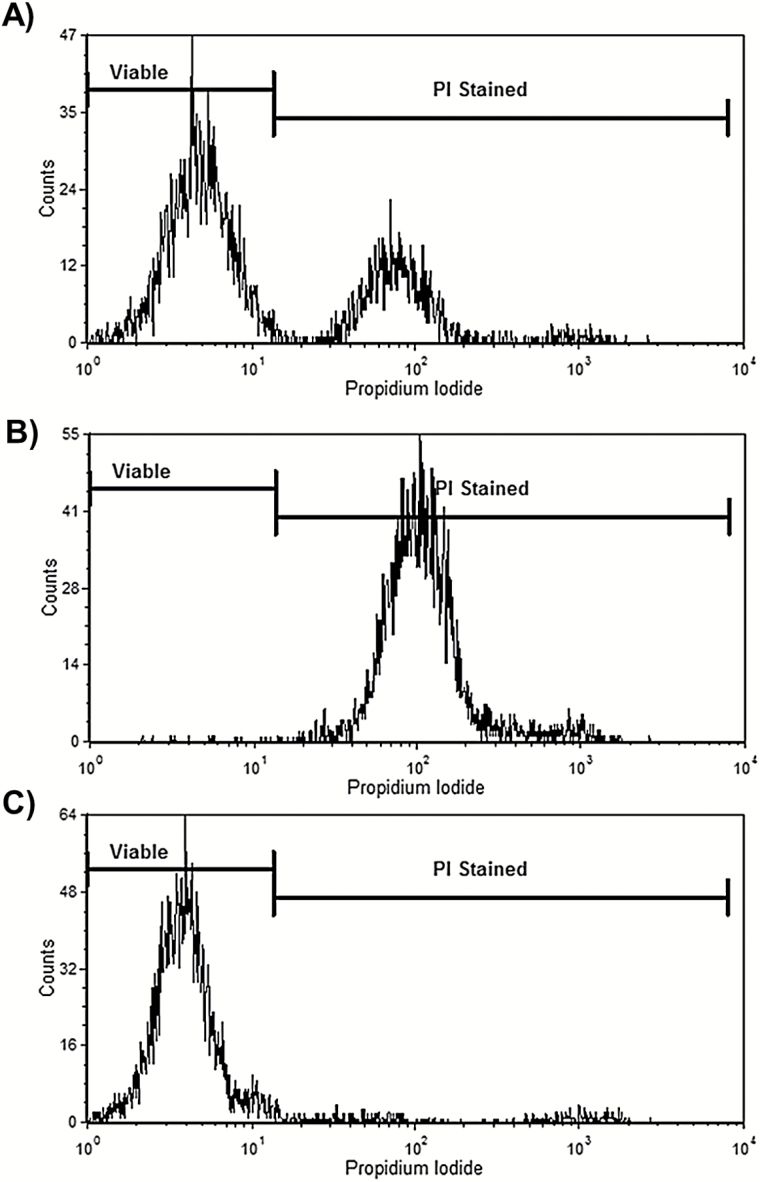
FCM analysis of human lymphoblastoid cells (TK6) following aerolysin and PI treatment. Flow cytometric outputs following consecutive aerolysin and PI treatment on parental TK6 strain (**A**) and FACS enriched CD59 positive (**B**) and negative populations (**C**).

When the parental CD59 heterogeneous TK6 cell line was treated with aerolysin and stained with the vital dye, PI, the viable cell population (PI-negative) is reflected in the frequency of cells that are negative for CD59 staining (Figure 2A). Aerolysin treatment of FACS-enriched CD59-positive cell cultures (i.e. presumptive *PIG-A* wild type) were predominantly PI-positive (Figure 2B), consistent with aerolysin permeabilisation at the GPI-anchor sites causing complete cell death. In contrast, aerolysin treatment of cell cultures derived from CD59-negative cells (i.e. presumptive *PIG-A* mutants) displayed negligible PI staining (Figure 2C), consistent with the absence of GPI-anchor sites expected of the *PIG-A* mutant phenotype.

From these studies, aerolysin treatment can also provide a means to directly select GPI-AP deficient cell clones and in turn, sufficient biological material for genomic sequencing of presumed *PIG-A* mutants (15,32,49,57).

Lack of cellular membrane integrity, characterised by the initial loss of membrane asymmetry, blebbing and the eventual formation of pyknotic bodies, is typically observed during mid-late stage apoptosis and subsequent cell death (58–60). Due to the extracellular localisation of GPI-AP, potential membrane alterations/inversion overserved during loss of membrane integrity could induce a misleading *PIG-A* mutant phenotype and hence, misleading conclusions regarding mutagenicity.

**Figure 3.**
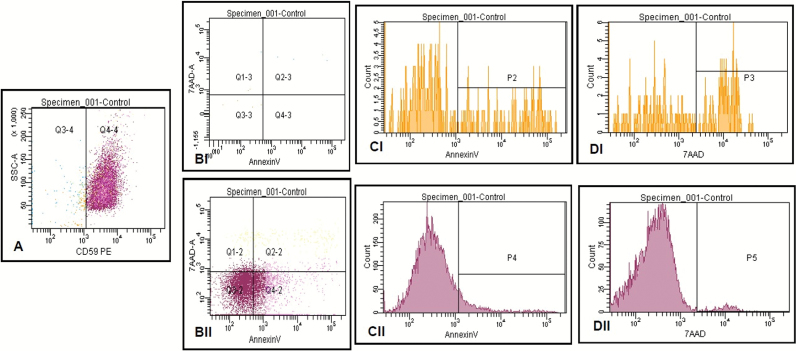
Preliminary validation of presumed phenotypic *PIG-A* mutant TK6 cells using flow cytometry. Viability assessment of clonally enriched CD59-R-PE–stained untreated TK6 cells (**A**) *PIG-A* mutant phenotype assessment, (**B**—I, II) viability dot plot, (**C**—I, II) apoptosis measure (Annexin V) and (**D**—I, II) loss of membrane integrity (7-AAD; ~10 000 single cellular events), *PIG-A* mutant phenotype and *PIG-A* wild-type cytograms denoted by I and II, respectively. P gates defined from maximum auto fluorescence observed following instrument calibration standard analysis within the respective channel.

To further characterise putative phenotypic *PIG-A* mutants, cells were stained with Annexin V Alexa Fluor 488 and 7-AAD to determine cell membrane integrity and apoptosis status (phosphatidyl serine presence). Of the TK6^CD59+^ cells (97.6%), 10.4% were Annexin V-positive (apoptotic) and 2.1% were 7-AAD-positive (lack of cellular membrane integrity). The small (~2%) CD59-negative cell population (i.e. phenotypic *PIG-A* mutants) showed much higher levels of apoptosis or loss of cellular membrane integrity (36.4 and 39.2%, respectively (Figure 3). Additional sampling, i.e. extended event capture, *n* = 500 000 events, confirmed this observation (Supplementary Figure 4, available at Mutagenesis Online).

These data suggest an uneven distribution of viable cells within the CD59-positive and CD59-negative cell populations, i.e. between the putative phenotypic *PIG-A* wild-type and mutant populations. Moreover, the high prevalence of non-viable cells in the CD59-negative cell population challenges the previous assumptions made about the frequency of presumptive *PIG-A* mutants. This could result in artefact, leading to elevated background frequencies of putative *PIG-A* mutants and a masking consequence if not fully removed from analysis; the overall effect could greatly decrease assay sensitivity (61).

### GPI-AP candidate trials in different human cell lines

The establishment of a promising assay design within TK6 cells led to the identification of a potential misinterpretation of phenotypic *PIG-A* mutant frequencies. In an attempt to alleviate the viability issue and hence, remove potential preparative stages that could influence viability, additional cell line and reporter phenotypic combinations were assessed in order to identify an unenriched low prevalence of spontaneous *PIG-A* mutants. The variability, in terms of surface antigen expression, between the different candidate human B-lymphoblastoid cell lines being considered for assay development were evaluated. Immunofluorescence staining was used to assess the expression of several well-characterised cellular surface antigens or the GPI-anchor status of the cell type, i.e. FLAER, B-lymphocyte activation marker [(BLAST-1)/(CD48)], protectin (CD59) and decay-accelerating factor (DAF/CD55).

FLAER is a fluorescently modified version of inactive aerolysin, retaining high affinity for GPI-AP moiety binding. Due to its comprehensive binding, FLAER was investigated for its use as a generic reporter for *PIG-A* mutation detection. CD48 was chosen for its GPI-AP characteristics and routine use as a B-lymphocyte activation marker (BLAST-1).

**Figure 4.**
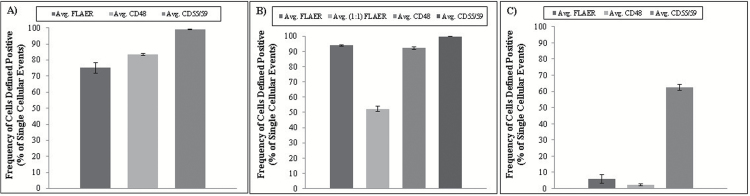
Average cellular surface marker expression within AHH-1, MCL-5 and low passage TK6 cells. FLAER–Alexa Fluor 488™ conjugate, CD48 phycoerythrin conjugate and CD55/59 phycoerythrin conjugate fluorescent dyes utilised to estimate surface marker expression. (**A**) AHH-1, (**B**) MCL-5 and (**C**) low passage TK6 cells (~10 000 single cellular events; *n* = 3, error bars ±1 SD).

Low passage TK6 cells were shown to stain poorly for all four cell surface antigen candidates, with only 5.9% cells positive for FLAER (at top concentration 2.5 µg/mL), 2.2% cells positive for CD48 expression and 62.6% cells positive for CD55 and CD59 expression (Figure 4). The AHH-1 cell line showed higher levels of staining, i.e. 75.2% cells were positive for FLAER staining, 83.5% cells positive for CD48 staining and 99.2% cells positive for CD55 and CD59 expression (Figure 4). MCL-5 exhibited the most comprehensive staining for all fout candidates, i.e. 93.5% cells positive for FLAER, 92.1% cells positive for CD48 and 99.8% cells positive for CD55 and CD59 (Figure 4).

The background levels of CD55 and CD59 expression in MCL-5 cells were similar to those of the original TK6 cell lines used following enrichment (TK6^CD59+/CD55+^). Given these fundamental properties and the potential for artefact induction following a loss in viability during enrichment, MCL-5 cells were chosen for the continuing development of the *in vitro PIG-A* assay.

### Viability assessment of MCL-5 Cells to avoid potential artefacts

P53 status and, hence, apoptosis functionality has been a debated topic in regard to the cell lines utilised within *in vitro* genetic toxicology testing (62,63). Therefore, prior to *PIG-A* assessment, a more detailed viability assessment was undertaken to establish apoptosis capability and provide evidence for p53 functionality. In previous TK6 cell experiments, a high prevalence of non-viable cells (apoptotic or necrotic) had been observed in the CD59-negative cell populations, which could confound an accurate estimate of the *PIG-A* mutant phenotype. Therefore, the impact of apoptosis on cell surface antigen expression in MCL-5 cells was assessed using cells treated with the apoptosis inducer staurosporine.

**Figure 5.**
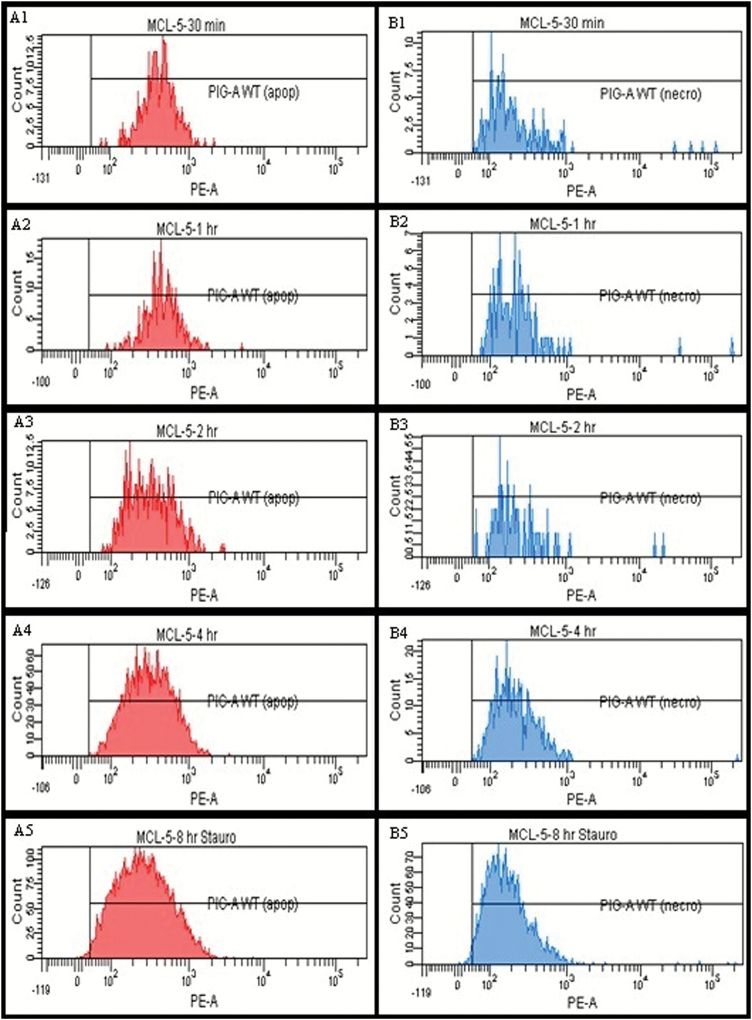
Tracking phenotypic *PIG-A* status through apoptosis and necrosis. Phenotypic *PIG-A* mutant frequency data for MCL-5 cells undergoing (**A**) apoptosis and (**B**) necrosis following a time course acute 1 µM staurosporine exposure: (1) 30 min, (2) 60 min, (3) 120 min, (4) 240 min and (5) 480 min. Relative PE detector signals utilised as the measure for surface GPI-AP expression (>3000 events analysed in each sample). ‘*PIG-A* WT’ gate present defines threshold for phenotypic *PIG-A* mutant status.

Cell viability was assessed at various time points using flow cytometric light scatter properties together with GPI-AP labelling and Annexin V and 7-AAD staining. Following staurosporine treatment, there was a dose- and time-dependent increase in the frequency of apoptotic cells and loss of cellular membrane integrity (Supplementary Figure 5, available at Mutagenesis Online); although at the earlier stages there was no significant change in cell morphology by FCM (determined by forward and side scatter profiles). Significantly, a corresponding decrease in GPI-AP expression (Figure 5) was also observed, with top doses seemingly inducing phenotypic *PIG-A* mutant events (Table 2).

**Table 2.** Tracking phenotypic *PIG-A* status through apoptosis and necrosis

Sample identification	Necrotic cells			Apoptotic cells		
	Percentage of dead cells	CD55/59- positive (%)	CD55/59- negative (%)	Percentage of viable cells	CD55/59- positive (%)	CD55/59- negative (%)
30 min	27.8	100.0	0	2.3	100.0	0
60 min	43.0	100.0	0	4.4	100.0	0
120 min	24.9	100.0	0	5.2	100.0	0
240 min	61.8	100.0	0	44.2	99.9	0.061
480 min	76.0	98.7	1.3	46.7	99.9	0.108

Phenotypic *PIG-A* mutant frequency data for MCL-5 cells undergoing (A) apoptosis and (B) necrosis following a time course acute 1 µM staurosporine exposure (>3000 events analysed in each sample).

Since the previous observations in TK6 cells were also seen with MCL-5 cells, there is clearly the potential for cell viability artefacts to confound the *PIG-A* mutation phenotype assessment. It is therefore critical that viability dyes (both apoptosis and cell death) and accurate/reproducible FCM gating are applied in the development and assessment of *in vitro PIG-A* assays.

### Determination of *PIG-A* mutant frequency using the CD59 phenotypic marker, viability assessment and HLA-DR staining within MCL-5 cells

**Figure 6.**
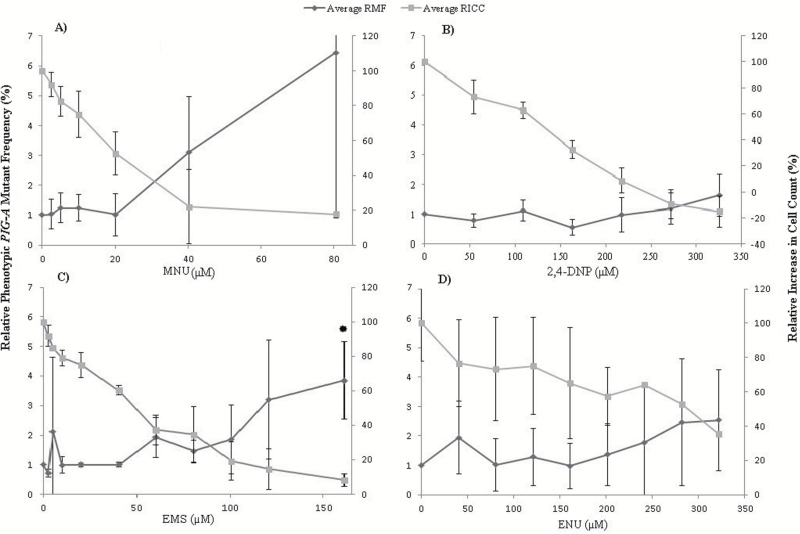
Trialled usage of HLA-DR staining to validate single marker *PIG-A* dose response data within MCL-5 Cells. Day 4, low passage MCL-5 average relative phenotypic *PIG-A* mutant frequency following 24 h (**A**) MNU (**B**) 2,4-DNP, (**C**) EMS and (**D**) ENU exposure, CD59 antigen utilised as reporter for mutation, inclusion of HLD-DR and 7-AAD staining to ensure cellular membrane integrity (Dunnett’s/Dunns test; *P < 0.05; *n* = 3; error Bars ±1 SD) *PIG-A* mutant data relative to the percentage of phenotypic *PIG-A* mutants observed within the concurrent control.

To evaluate the possible benefit in phenotypic *PIG-A* mutation detection accuracy, transmembranous membrane protein marker staining was trialled to more accurately exclude non-viable events from FCM analysis. HLA-DR was chosen as a trial membrane marker for its comprehensive expression within the cell line utilised and to provide additional confidence in phenotypic identity. To investigate the utility of MCL-5 cells in the *in vitro PIG-A* assay, cell cultures were exposed to three alkylating agents, as positive mutagens, i.e. EMS, MNU or ENU and as a negative control, 2,4 DNP. Following 24 h treatment, cells were harvested and subject to daily modified cell staining including anti-HLA-DR (membrane integrity), 7-AAD and anti-CD59 to determine *PIG-A* phenotype (Figure 6).

Acute MNU exposure resulted in a dose-dependent increase in the frequency of viable CD59-negative MCL-5 cells, i.e. mutant *PIG-A* phenotype, from 1.01% to 6.43% at the top concentration tested. Although there was a strong positive correlation between dose, cytotoxicity and phenotypic *PIG-A* mutants, none of the doses were statistically significant (*P* > 0.05). EMS exposure resulted in a comparable dose-dependent increase, with the top dose of 161 µM (equivalent to ~20 µg/mL) eliciting a statistically significant increase (*P* < 0.05) with a corresponding increase in cytotoxicity [8% relative increase in cell count (RICC)]. ENU exposure did not increase the number of CD59 negatively stained cells over background, i.e. the relative average phenotypic mutant frequency (*P* > 0.05). However, data did appear to be affected by variance between replicates, and cytotoxicity again was observed to be elevated. Acute 2,4 DNP exposure resulted in a non-linear Day 4 dose response, with no evidence for any correlation between dose and increase in relative *PIG-A* mutant frequency (*P* > 0.05). Cytotoxicity again was demonstrated to be highly dose dependent, with top doses eliciting significantly increased cytotoxicity, however, with no corresponding increments of *PIG-A* mutant frequency (as expected).

The data demonstrated the expected EMS and MNU results in terms of potency, MNU was observed to induce the larger fold increase in CD59-negative cells, over the concurrent controls. However, ENU did not induce the expected dose response most likely due to excessive variation within replicates. Utilising significantly shorter assay durations (4–5 days) than those recently described in analogous publications (8–14 days) (50,64) may contribute to the reduced observed genotoxic effect of ENU, possibly preventing an adequate time period for mutant phenotype manifestation. However, like 2,4 DNP, ENU showed little evidence for a positive correlation between cytotoxicity and elevated numbers of CD59 negatively stained cells, i.e. phenotypic *PIG-A* mutant frequency. This suggests that the inclusion of membrane assurance markers lessened the potential induction of phenotypic *PIG-A* mutant mimicking events. 2,4 DNP, a non-genotoxicant, was demonstrated to not increase phenotypic *PIG-A* mutant frequency across the entire dose range, even at doses that elicited severe cytotoxicity.

### Tandem GPI-AP confocal expression analysis in MCL-5 cells

Limited physiological data, in regard to the *Pig-a* mechanism, is available to describe the nature of extracellular surface protein localisation and density on the surface of human lymphoblastoid cells. Within the scope of the *PIG-A* assay, mutagenesis is indirectly measured through the presence or the absence of GPI-AP, and therefore, mechanistic biological evidence at the cellular level could be used to provide increased integrity. Therefore, the localisation, density and coverage of initially single and sequentially tandem GPI-AP markers were investigated on the surface of MCL-5 cells using high-resolution confocal image analysis.

The proteins CD58, CD55 and CD59 were identified as possible cell surface biomarkers for the *PIG-A* phenotypic mutation. CD58 is a well-characterised GPI-AP, integral in strengthening the interaction between T cells and macrophages (65) and is expressed at intermediate/high levels on most subsets of normal peripheral blood leucocytes (66). CD59 (protectin) and CD55 (DAF) are used routinely for clinical diagnostic screening of PNH, associated with *PIG-A* mutation (19), and CD59 is also used in the *in vivo Pig-a* assay in rodents (29,32,34,67). However, CD58 was later demonstrated to have a transmembranous single-pass isoform (65) and therefore, CD59 and CD55 were selected for further evaluation in the development of this *in vitro* assay design.

**Figure 7.**
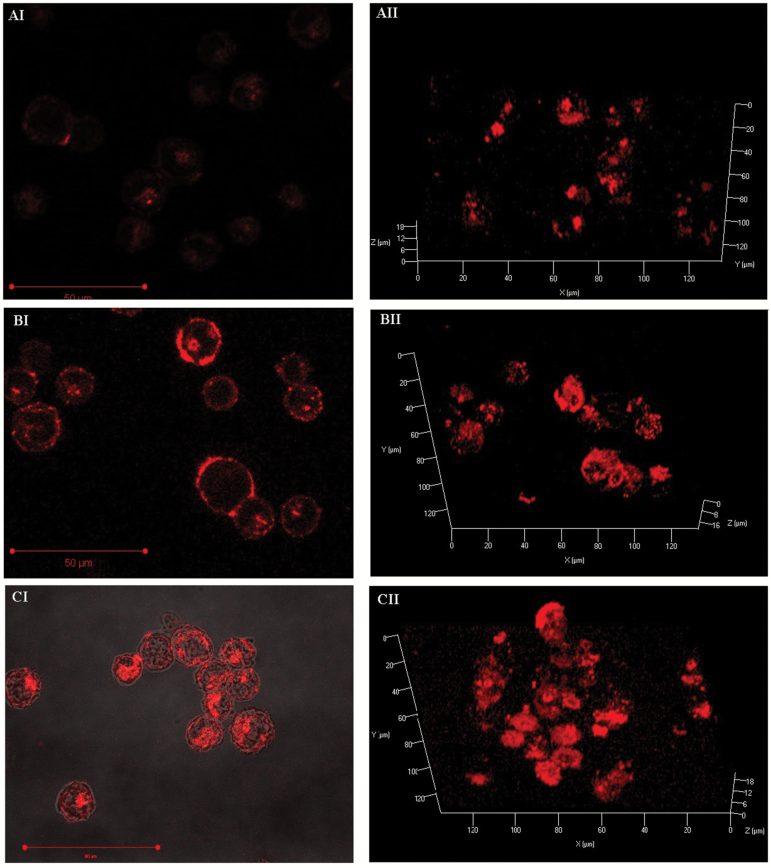
GPI-AP surface expression analysis within MCL-5 cells. Utilising either or both anti-CD55 R-PE/CD59 R-PE treated 4% PFA fixed MCL-5 cells, confocal microscopy images were generated under the 63X oil immersion objective. High resolution images were produced and displayed within a 3-D reconstruction following 1024 × 1024, z-stack capture utilising the laser scanning confocal microscope. Detected, R-PE signal was retrospectively artificially coloured for image clarity and cohesion (scale bar 50 µM).

Results indicated that both CD55 and CD59 immunofluorescence staining was associated comprehensively with the extracellular membrane localisation (Figure 7 AI and BI) as indicated by a distinctive halo image, reflecting the presence of peripheral cell staining. Utilsing 3-D reconstructed Z-stacked images, single GPI-AP expression was clearly defined as punctate in nature and consisted mainly of dense pockets of cell membrane expression (Figure 7 AII and BII). CD59 surface antigen expression was estimated to be less comprehensive than CD55. There was no clear association/correlation between staining density and distinct morphological features in the membrane following the construction of a composite image utilising transmitted light (Figure 7 CI). However, tandem labelling with the combined CD55/59 generated significantly greater staining and surface coverage (Figure 7 CII); and although the antibodies were still localised to densely staining regions, a much larger proportion of the extracellular membrane was visible. The punctate nature of staining was identified as a potenital limiting factor when utilsing specific excitatory sources and subsquent fluorescent image capture sysytems.

Utilsing CD55/59 tandem labelling to determine *PIG-A* phenotype facilitates greater confidence in the definition of presumptive *PIG-A* mutant cells. The unlikely probability of losing both surface antigen signals through a non-mutagenic mechanism during analysis significantly reduces the likelihood of misleading results when combined with appropriate cell viability labelling. In addition, due to the punctate nature of GPI-AP association with the surface membrane, and the nature of laser light excitation and recording, specific laser platforms could suffer from a bias due to the plane of light excitation and hence surface marker exposure.

### Gating strategy investigations

Assay refinement and cell line alterations have led to the development of an *in vitro PIG-A* assay design utilising: (i) a membrane assurance marker (HLA-DR); (ii) viability markers (Annexin V and 7-AAD) and (iii), tandem antibody reporting markers (CD55/59). However, gating strategy also contributes significantly to the performance of the analysis. The use of alternative properties of the recorded light signal, other than solely the average or maximum fluorescence value generated, was investigated to more accurately define a viable single-cell population in which to assess *PIG-A* phenotype. Fluorescence width measurements (time of flight) have been recently incorporated into FCM analysis as an additional variable to be used to estimate cell size and therefore, offer a potential supplementary layer of cell characterisation during analysis.

**Figure 8.**
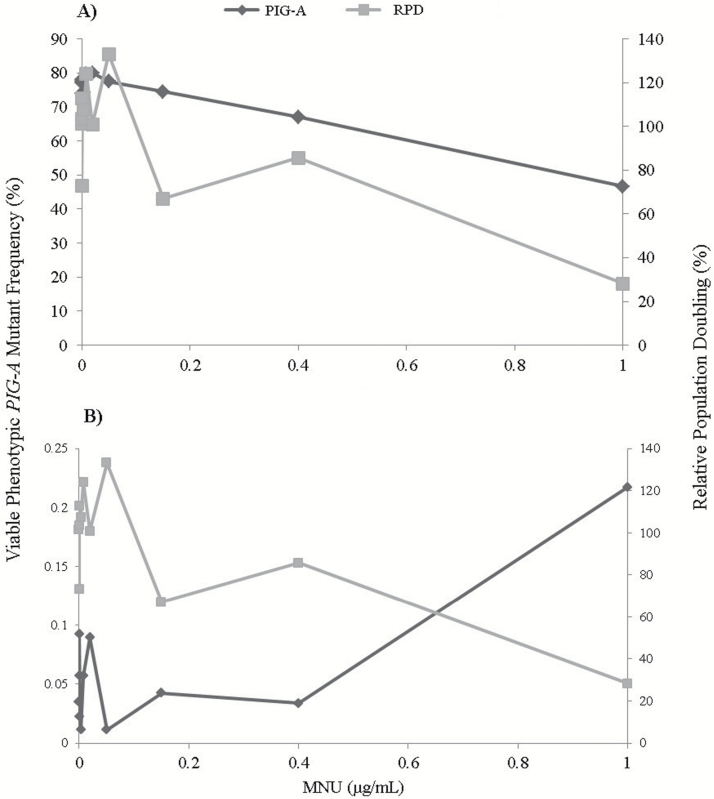
Establishment of a robust in vitro *PIG-A* assay gating strategy. Utilising viability dye incorporation (Annexin V and 7-AAD) and tandem markers (CD55 and CD59) the proposed robust reproducible gating strategy (**B**) was directly compared against traditional flow cytometry gating (**A**). Retrospectively, Day 3, 24 h MNU (0–1 µg/mL) exposure data within MCL-5 cells was assessed using both methods in order to estimate phenotypic *PIG-A* mutant frequency and hence, dose response.

Following the trialling of three gating strategies (Table 3), each utilising a different combination of fluorescence area and width measurements to define the single-cell population; it was apparent that the strategy had significant implications on the final presumptive *PIG-A* mutant frequency. Utilising pulse width measurements, analogous to that observed within a typical doublet discriminant plot, as an additional means to classify particles as single cell in nature could significantly reduce the number of events falsely being defined as single cell and therefore more accurately define the phenotypic *PIG-A* mutant frequency. Utilising a retrospective analysis on singlet Day 3, 24-h MNU exposure data within MCL-5 cells (Figure 8), more realistic mutant frequency data (~100 phenotypic mutants per 1 × 10^6^ cells scored) was generated following the implementation of the refined gating strategy. However, pulse width measurements in combination with area measures for the fluorophore reporting phenotypic *PIG-A* status can have a biasing effect on mutant gating, when restrictive region gates were applied to the fluorescence area axis. This gating combination underestimated the number of defined phenotypic *PIG-A* mutant cells, unless gating counter measures were used.

**Table 3.** Summary of presumed phenotypic *PIG-A* status of parental untreated MCL-5 cells, pre-enrichment utilising different gating strategies.

Sample ID	Number of recorded events	Single-cell events	Trialled gated events	Number of viable cells	CD55/59-positive (%)
Instrument calibration standard	43 856	20 575	17 574	17 573	0
Original gating	48 629	20 569	n/a	17 577	85.45
Trialled gating 1					
FSC-A vs PE-W 1	48 629	20 391	18 056	17 874	99.99
FSC-A vs PE-W 2	59 135	20 495	17 983	17 750	99.99
FSC-A vs PE-W 3	48 190	20 418	18 171	18 019	100.00
Average	51984.7	20 434.7	18 070	17 881	99.99
Trialled gating 2					
PE-A vs PE-W 1	48 629	20 841	18 584	18 407	100.00
PE-A vs PE-W 2	59 135	20 882	18 384	18 140	100.00
PE-A vs PE-W 3	48 190	20 748	18 431	18 245	100.00
Average	51984.7	20823.7	18466.3	18 264	100.00

Tandem surface antigen markers CD55 and CD59 were utilised as indirect reporters of *PIG-A* mutation in order to assess phenotypic *PIG-A* mutant status following incorporation of pulse width measurement gating strategies (>20 000 defined single cellular events were captured).

FCM analysis can be subjective without the use of standardised protocols. Therefore, identifying reproducible methods for establishing thresholds and utilising unbiased gating approaches are fundamental in establishing comprehensive test systems with confidence and accuracy. Utilising pulse width measures in combination with a robust threshold gating strategy could significantly reduce the number of falsely identified putative phenotypic *PIG-A* mutant events (Figure 8). Doublet discrimination and correct pulse width measures more accurately define the viable single-cell population and therefore can prevent the inclusion of doublets, underestimating/overestimating phenotypic mutant frequency, or debris from inducing false positives. Standardising the gating approaches and further refining assay design has been shown to reliably reduce artificially high baseline mutant frequencies (~5–50 × 10^−6^ mutants) and has brought the perceived assay sensitivity more in line with currently validated *in vitro* gene mutation endpoints (MLA/HRPT), enabling future low-dose investigations and potential quantitative dose–response modelling.

## Conclusions

During this study, the proposed *in vitro PIG-A* assay design underwent a number of revisions and refinements following identification of potential confounding factors. The assay design, established within the metabolically active, genetically modified, human MCL-5 cell line, has demonstrated proofs of principle and the potential focus for future work. Toxicity was demonstrated to be a continuous problem associated with the misidentification of the putative *PIG-A* deficient phenotype; however, the studies reported here suggest that these issues are effectively addressed through the inclusion of membrane integrity markers such as HLA-DR or alternatively CD19 (50) (highlighted by the 2,4-DNP and ENU data sets; Figure 6) in combination with adequate apoptosis and cell death markers.

Toxicity measures are used to define the doses tested and relevance of results within any mutation assay. A measure utilised within the *in vitro PIG-A* assay design which does not account for clonal expansion/cloning efficiency could miscalculate toxicity. RICC, RPD and relative cell counts are all measures that may not be strictly suitable outside of intended usage within the MN assay based on the duration of the assay. Shifting the toxicity measure more in line with analogous test systems, such as HPRT and/or MLA, could potentially alleviate toxicity misinterpretation, seemingly more apparent at the initial stages of phenotype assessment and could facilitate minimal experimental duration.

The genotype/phenotype relationship validation is still ongoing, recent publications reporting novel *PIG-L* gene mutations, following loss of heterozygosity deletions within the TK6 cell line, which also contribute to the GPI-AP deficient phenotype in addition to *PIG-A* mutations (38,64), have heightened the need for cell line-specific identification of additional genes active within the current phenotypic *PIG-A* scoring mechanism. Validatory sequencing experiments should not utilise any inadvertently biasing means of loss of phenotypic mutant selection if population statistics are to be generated and focus on whole genome sequence analysis or at least multiple *PIG* genes from the accurately gated loss of phenotype population.

In conclusion, this study suggests that a stand-alone *PIG-A* assay within MCL-5 cells is more promising than a TK6 cell-based assay. Further refinement, followed by a comprehensive assessment of specificity, sensitivity and transferability could lead to the establishment of a high content, high-throughput assay system with the potential for use within a screening as well as a regulatory hazard and risk assessment environment. All data from which conclusions were drawn is contained within the article or clearly referenced.

## Supplementary data

Supplementary Figures 1 to 5 are available at *Mutagenesis* Online.

## Funding

Ph.D. work of B.R. was supported by the Engineering and Physical Sciences Research Council–GlaxoSmithKline case award [EP/J502248/1]. Work carried out at Gentronix Ltd was funded by Gentronix Ltd.

## Supplementary Material

Supplementary Figure S1

Supplementary Figure S2

Supplementary Figure S3

Supplementary Figure S4

Supplementary Figure S5

Supplementary Figure Legends
