# Supplementary figures and images for: Development of an in vitro PIG-A gene mutation assay in human cells
Source: Mutagenesis. 2017 Jan 5;32(2):283–97. doi: 10.1093/mutage/gew059 (PMC5907909; doi:10.1093/mutage/gew059)

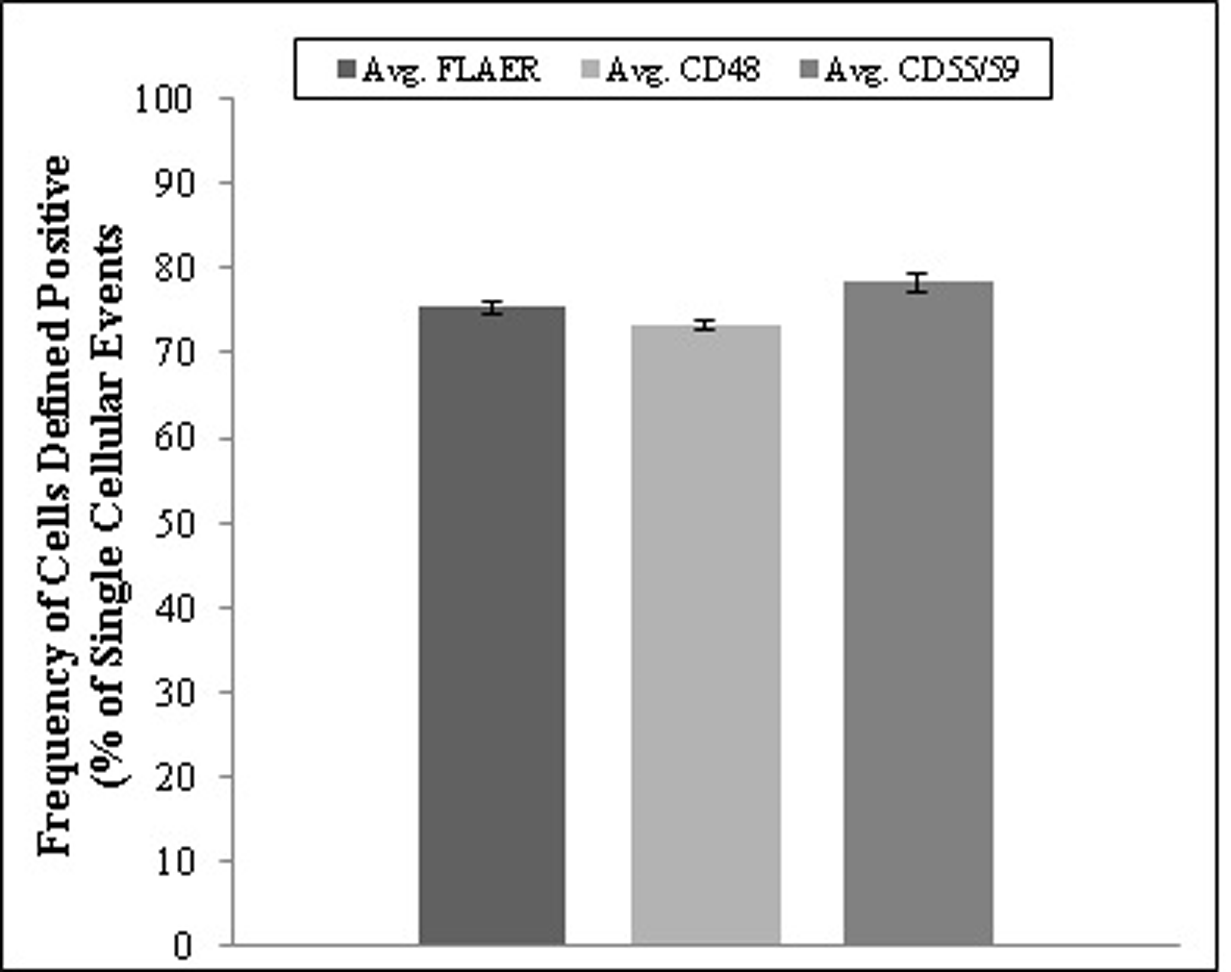

Supplement: Supplementary Figure S1 [file gew059_suppl_supplementary_figure_s1.png]

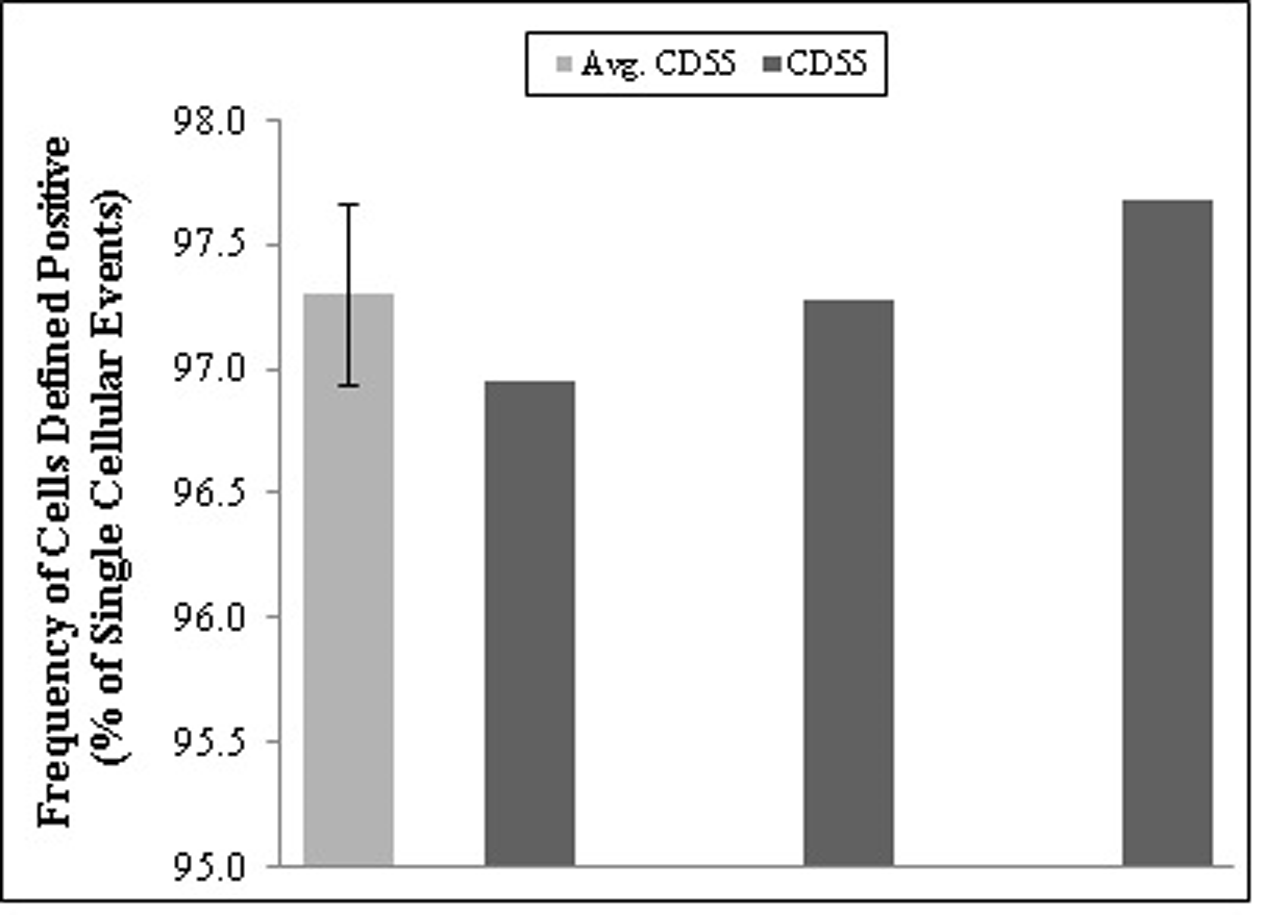

Supplement: Supplementary Figure S2 [file gew059_suppl_supplementary_figure_s2.png]

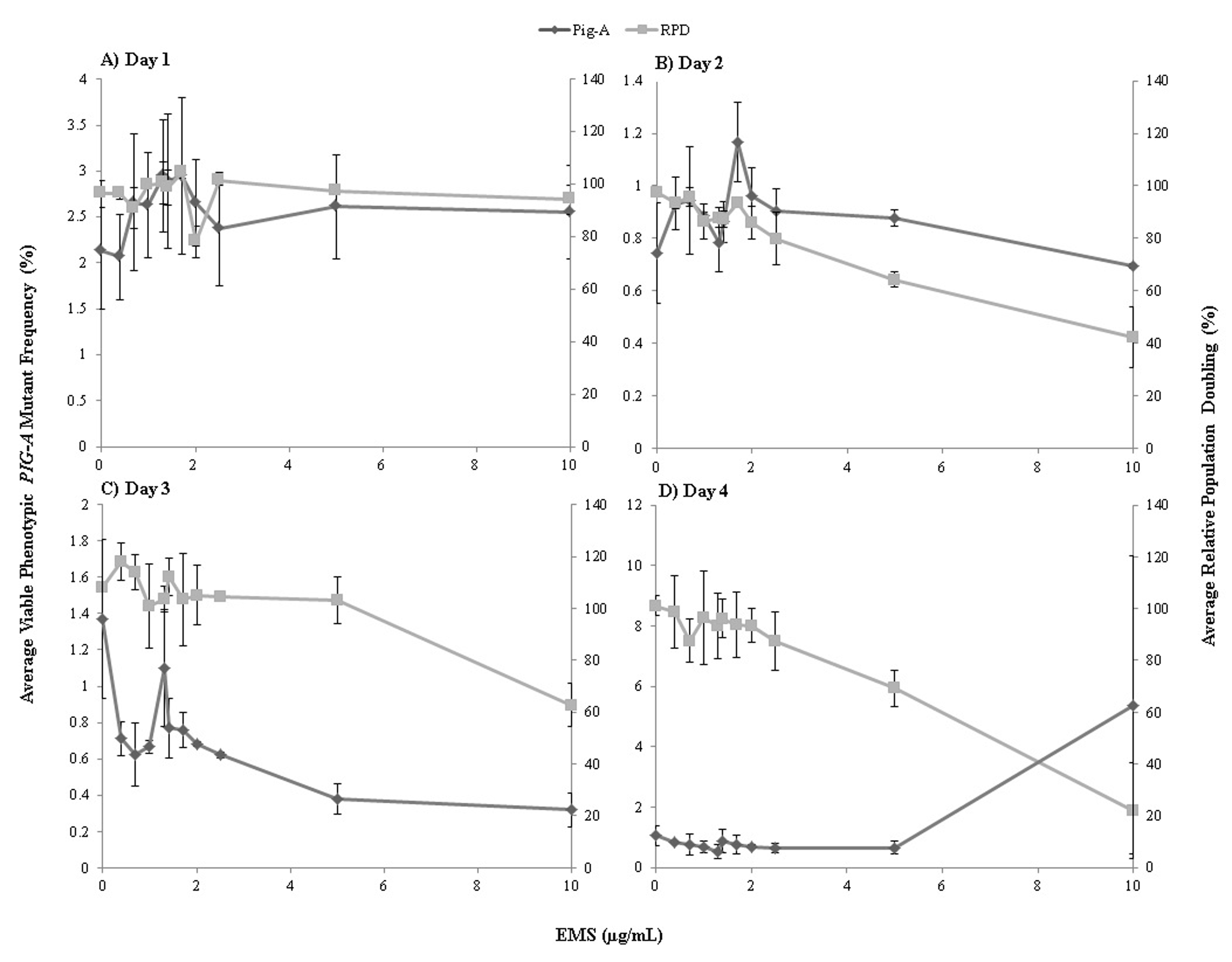

Supplement: Supplementary Figure S3 [file gew059_suppl_supplementary_figure_s3.png]

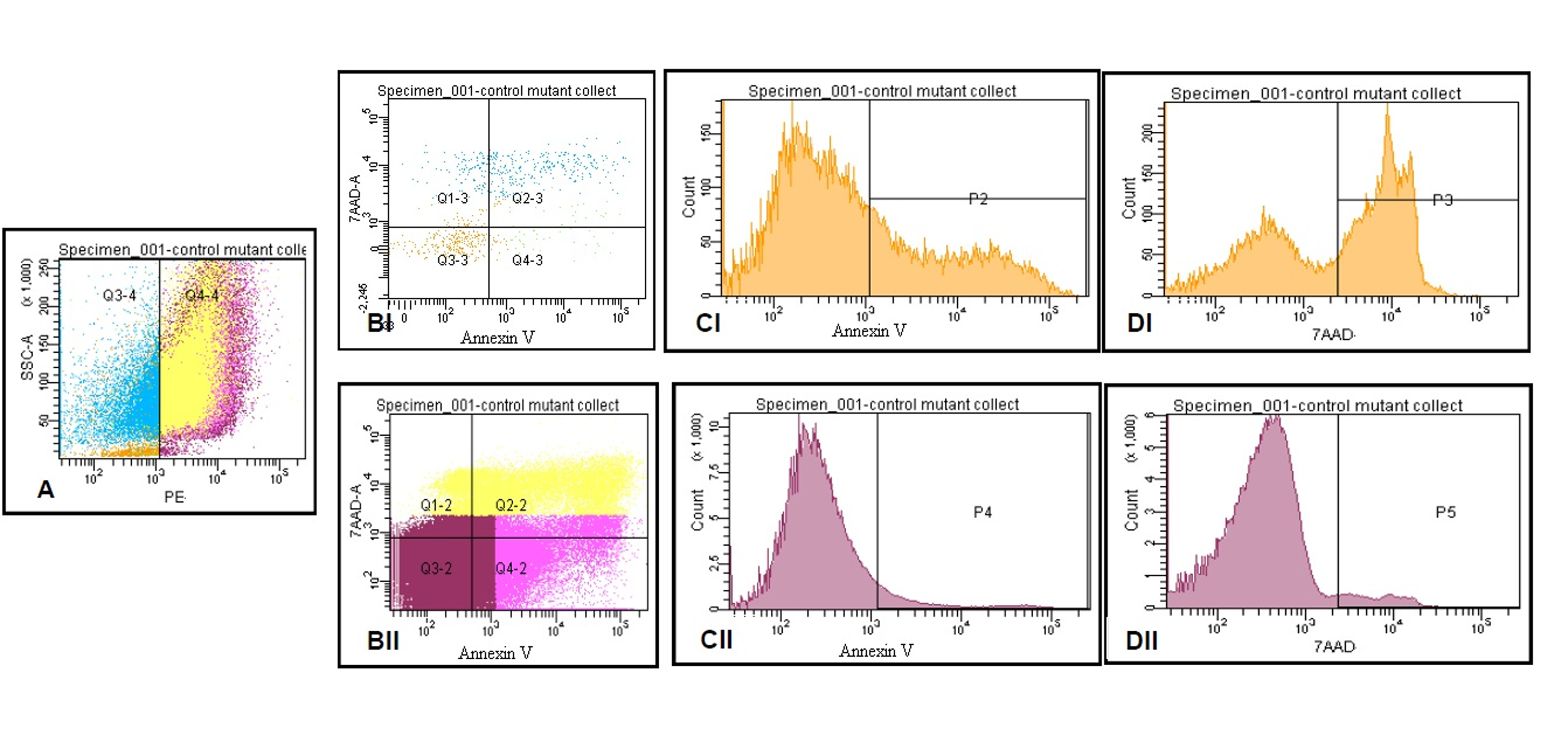

Supplement: Supplementary Figure S4 [file gew059_suppl_supplementary_figure_s4.png]

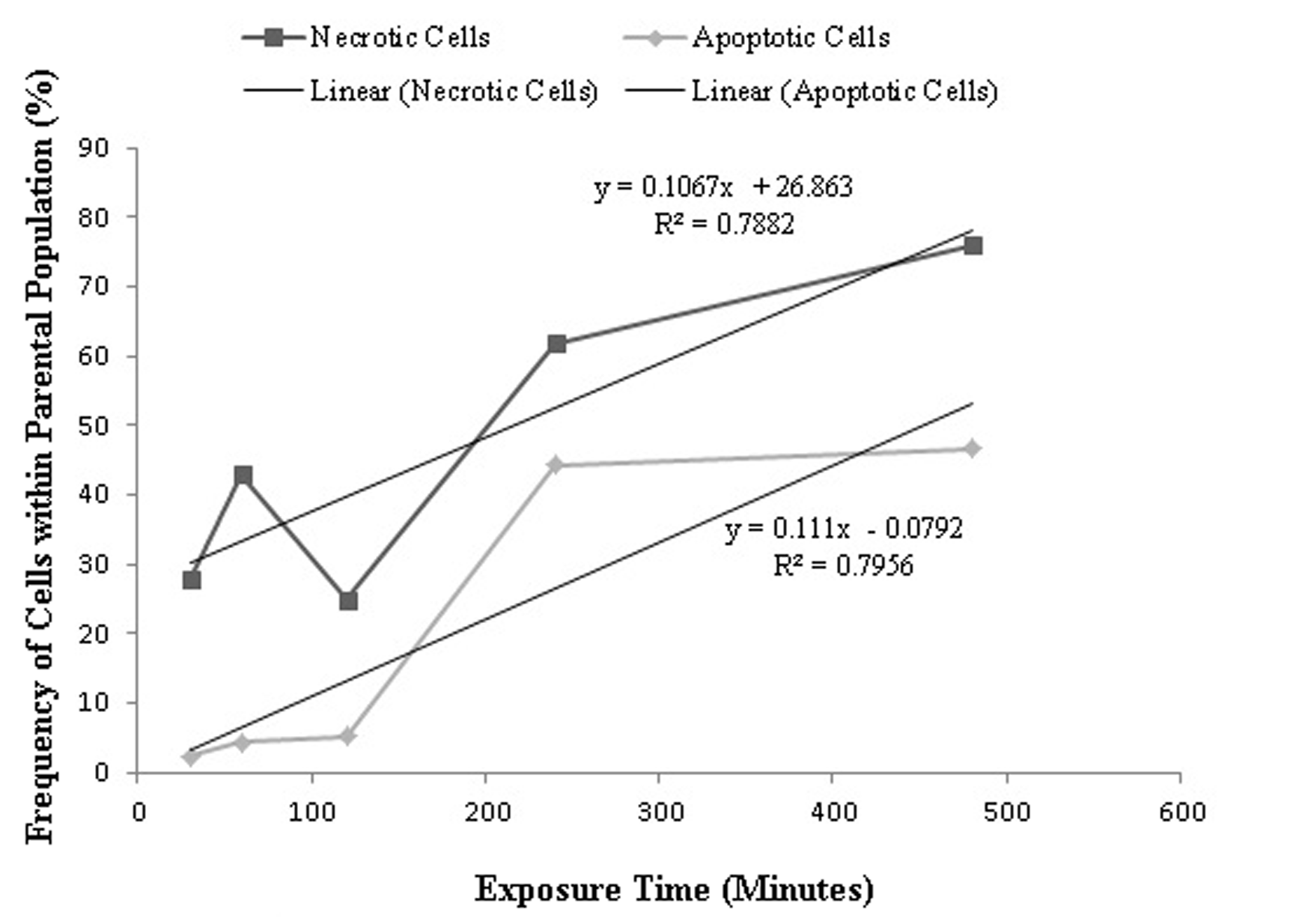

Supplement: Supplementary Figure S5 [file gew059_suppl_supplementary_figure_s5.png]
