# Supplementary material for: Development of an in vitro PIG-A gene mutation assay in human cells
Source: Mutagenesis. 2017 Jan 5;32(2):283–97. doi: 10.1093/mutage/gew059 (PMC5907909; doi:10.1093/mutage/gew059)
Supplement: Supplementary Figure Legends [file gew059_suppl_supplementary_figure_legends.docx]

**Supplementary Figure Legends**

**Supplementary Figure S1.** Average cellular surface marker expression within TK6 cells. FLAER (2.5µg/mL)–Alexa Fluor 488™ conjugate, CD48 phycoerythrin (R-PE) conjugate and CD55/59 phycoerythrin conjugate fluorescent dyes utilised to estimate surface marker expression (~10,000 single cellular events) (N=3, Error Bars ±1SD).

**Supplementary Figure S2.** Average cellular CD55 surface marker expression within Low Passage TK6 cells. Anti-CD55 R-PE staining was utilised in combination with flow cytometry to estimate cellular surface expression within Low Passage TK6 cells. Detector sensitivity was established following ICS analysis (N=3, Error Bars ±1SD)

**Supplementary Figure S3.** Low Passage TK6 preliminery *PIG-A* Assessment Utilising the CD55 Surface Antigen following 24hr EMS Exposure. Days 1-4 frequencies of FACS enriched phenotypic *PIG-A* mutant Low Passage TK6 cells following 24 hr low dose EMS exposure, CD55 antigen utilised as reporter of mutation (Dunn’s Test (p<0.05*)) (n=2, Error Bars ±SEM) (0.1% *PIG-A* Mutants = 1 mutant x 10^-3^ cells) – Day 4, top dose a single replicate collected 500 events.

**Supplementary Figure S4.** Extended validation of phenotypic *PIG-A* mutant TK6 cells. Extended viability assessment for clonally enriched anti-CD59 R-PE stained untreated TK6 cells A) *PIG-A* Mutant phenotype, BI,II) Viability Dot Plot, CI,II) Apoptosis measure (Annexin V) and DI,II) Loss of Membrane Integrity (7-AAD)(~500,000 Single cellular events), *PIG-A* mutant phenotype and PIG-A wild type cytograms denoted by I and II respectively. P gates defined from maximum auto fluorescence observed following ICS analysis within the respective channel.

**Supplementary Figure S5.** Percentage of necrotic and apoptotic MCL-5 cells following staurosporine exposure. Relative percentage of necrotic and apoptotic cells in relation to 1µM staurosporine exposure time, within MCL-5 cell cultures. Linear trend lines calculated and R^2^ values for both data sets to reflect the correlation within the cellular viability and duration of genotoxin exposure (>6,000 events scored for each time period).
